# Supplementary material for: Analysis of the Bacterial Microbiota in Wild Populations of Prickly Pear Cochineal, Dactylopius opuntiae in Morocco
Source: Insects. 2025 Nov 21;16(12):1184. doi: 10.3390/insects16121184 (PMC12733486; doi:10.3390/insects16121184)
Supplement: Supplementary file 1 [file insects-16-01184-s001.zip › Supp1_Remmal_et_all_final.pdf]

Supplementary Materials

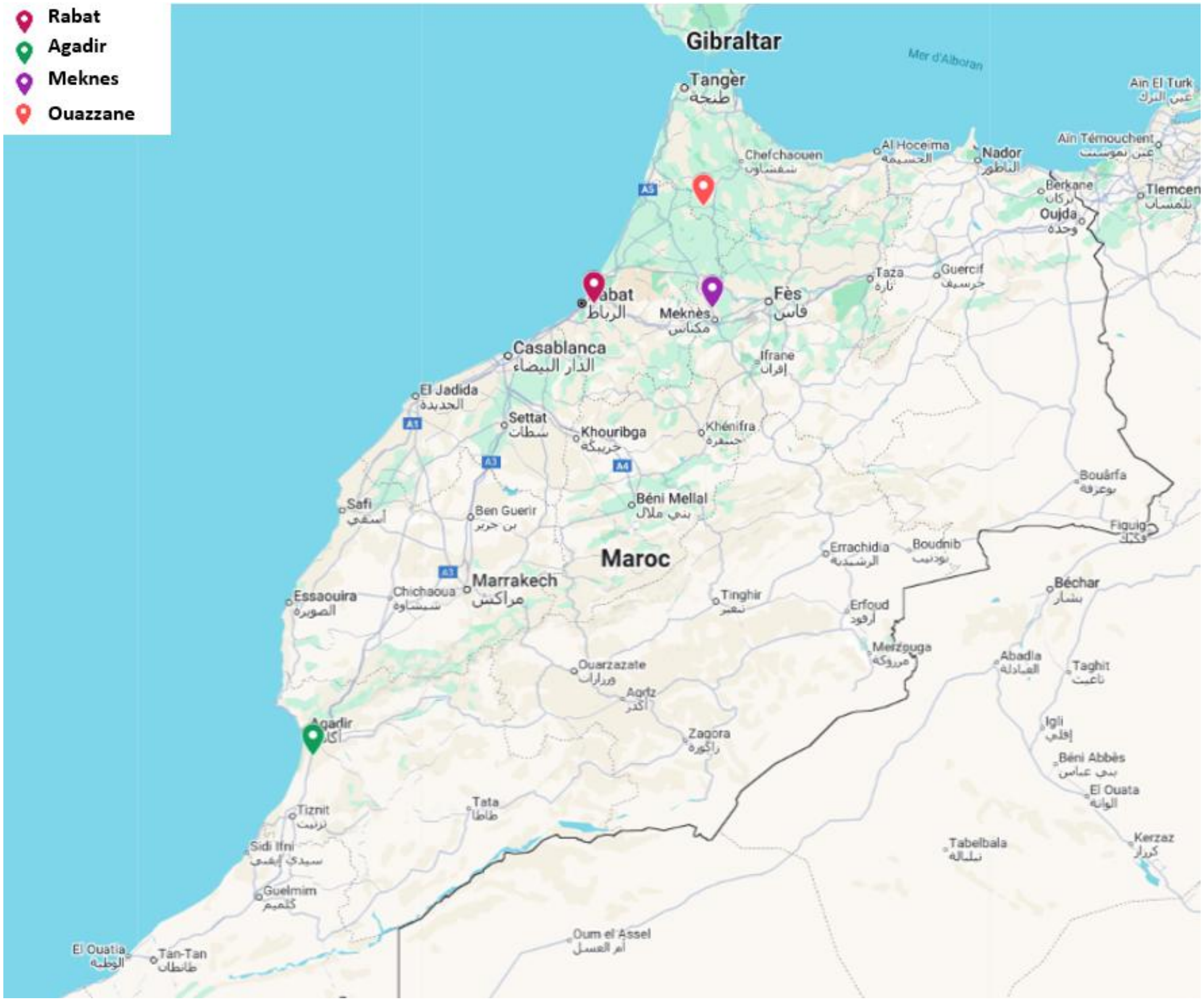

Figure S1. Graphical map of the regions

Table S1. List of bacterial primers and annealing temperatures

Gene	Primer	Sequence (5′-3′)	Tm (°C)	Product size
Wolbachia	WspecF	YATACCTATTCGAAGGGATAG	54 °C	438 bp
	WspecR	AGCTTCGAGTGAAACCAATTC		
Spiroplasma	SpouR1	CTTCAGTGTGCAGCTAACGC	55 °C	400 bp
	SpouF1	AAATCTTGTTAAGCAAGAAG		
**16S rRNA**	27F	AGAGTTTGATCCTGGCTCAG	54 °C	1500bp
	1429R	GGTTACCTTGTTACGACTT		

Table S2. Prevalence of bacterial endosymbionts screened in populations of *D. opuntiae*.
+ infected individuals (the number of infected samples per location), − uninfected individuals.

Population	Gender	Sample Size	*Wolbachia*	*Spiroplasma*
**Agadir**	Female	10	+(1)	**-**
	Male	10	-	**3**
	Nymph	10	-	**-**
**Rabat**	Female	10	-	**-**
	Male	10	-	**-**
	Nymph	10	-	**-**
**Meknes**	Female	10	+(1)	**-**
	Male	10	-	**-**
	Nymph	10	-	**-**
**Ouazzane**	Female	10	+(4)	**-**
	Male	10	+(6)	**1**
	Nymph	10	+(6)	**-**
**Total**		120	+(18)	+(4)

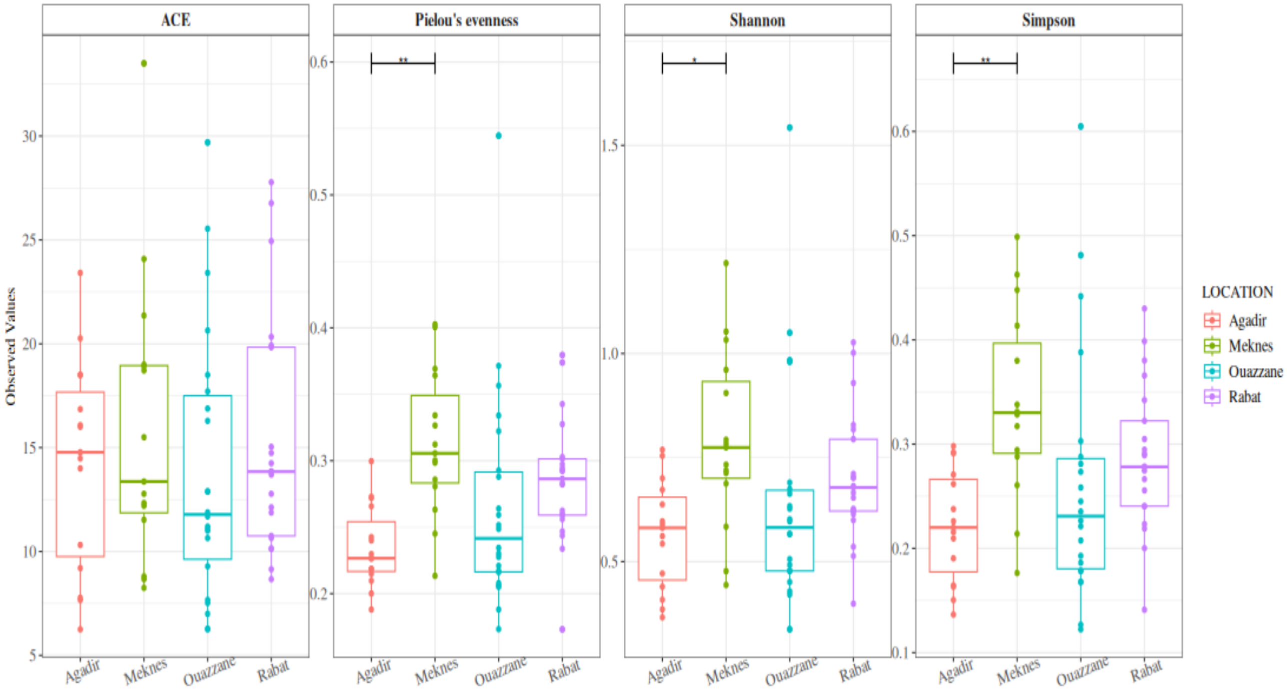

Figure S2. Species richness and diversity indices of *D. opuntiae* samples based on geographical location. Boxes indicate the inter-quartile range (IQR), the line within the boxes indicates the median, and the dots signify samples. (* $0.01 < p < 0.05$, ** $p \leq 0.01$).

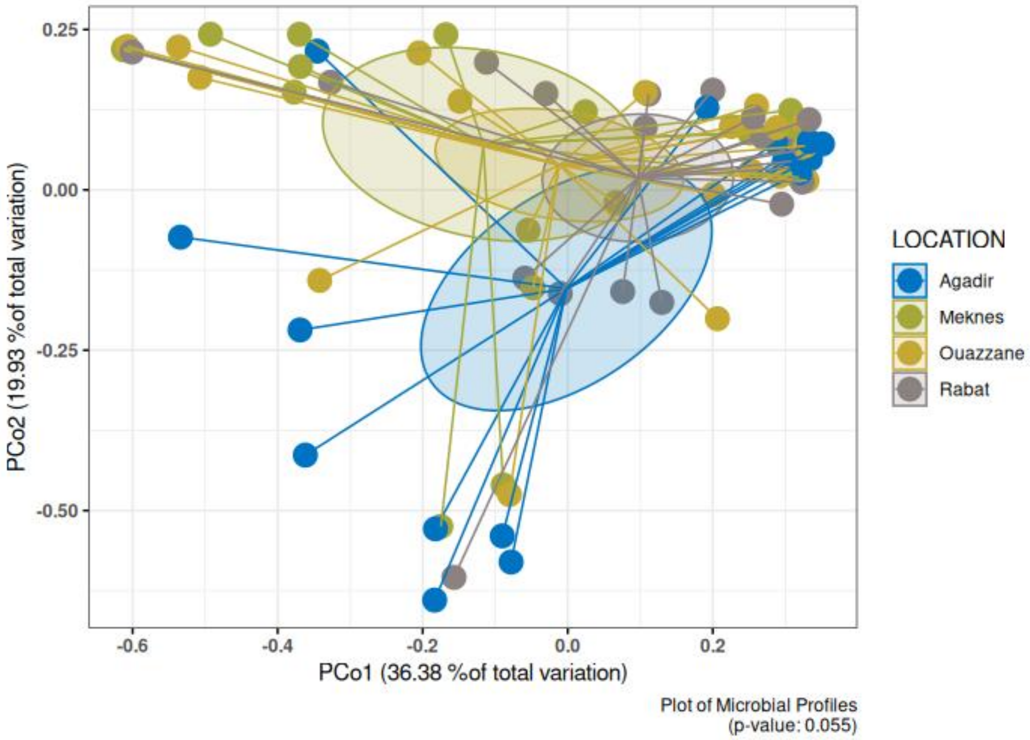

Figure S3. PCoA of *D. opuntiae*-associated bacterial communities, excluding *Candidatus* Dactylopiibacterium, according to the Bray-Curtis metric.

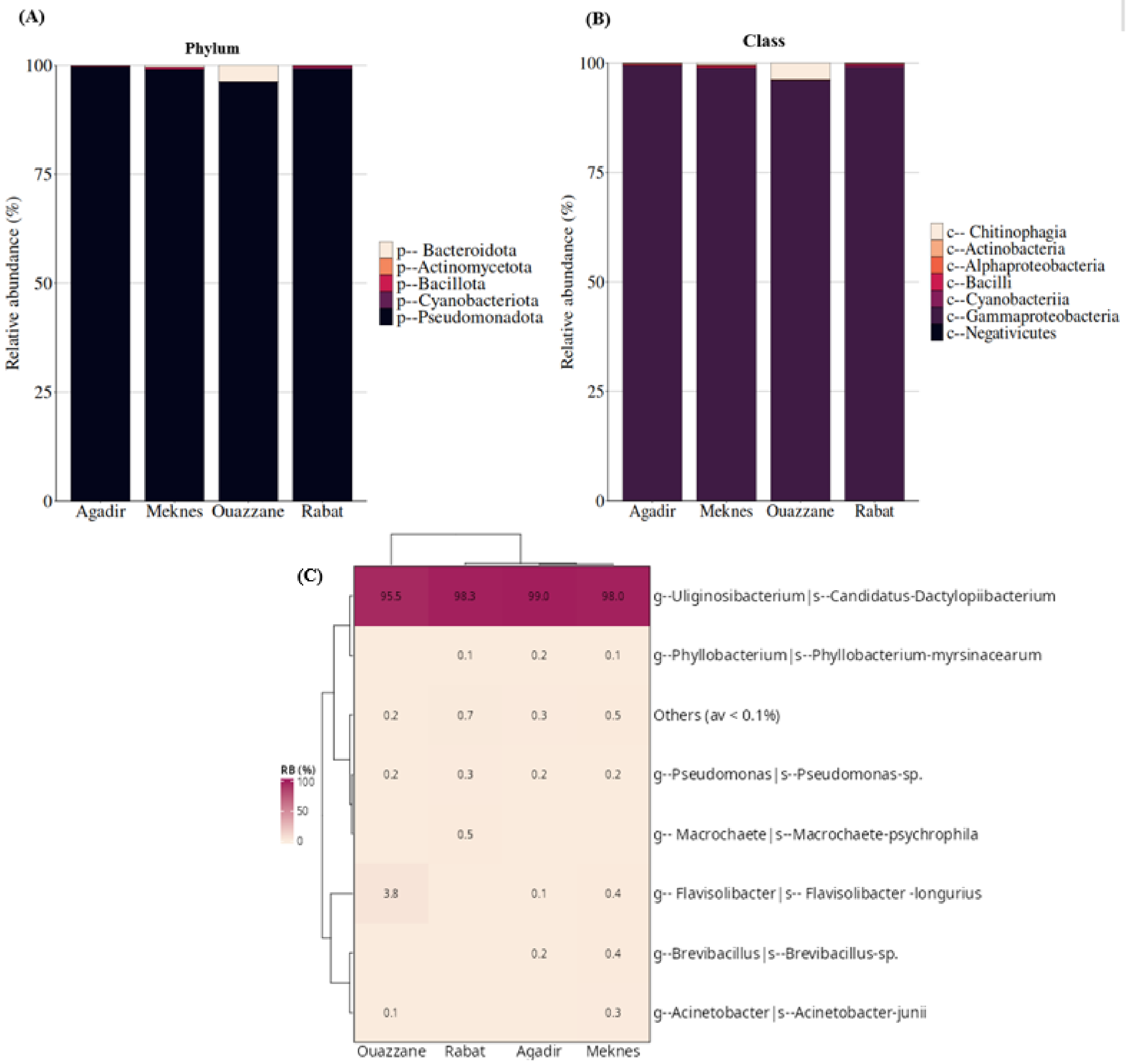

Figure S4. Relative abundance of natural *D. opuntiae* population microbiota at the phylum (A), class (B), and genus/species (C) levels.

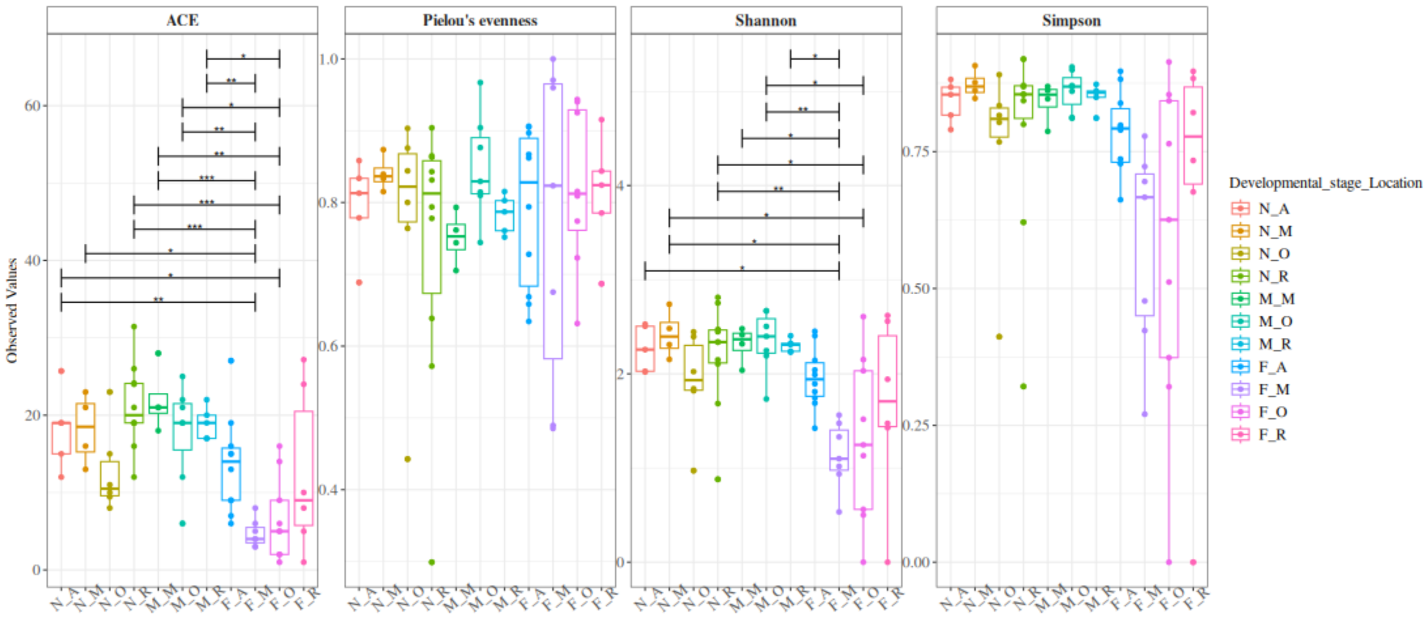

Figure S5. Species richness and diversity indices for *D. opuntiae* samples showed significant variations after excluding *Candidatus* Dactylopiibacterium based on their developmental stages and locations. The inter-quartile range (IQR) is shown by boxes, the median is shown by the line inside the boxes, and samples are shown by the dots. ** p<0.01, *** p<0.001, and * p<0.05.

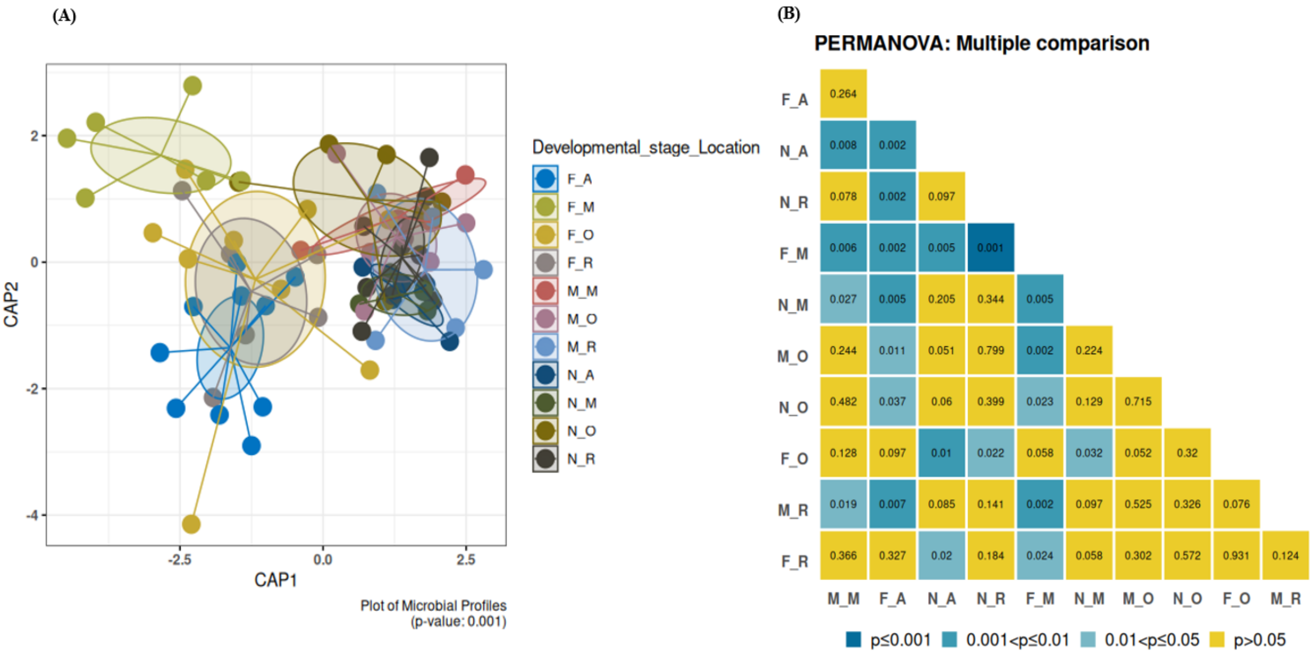

Figure S6. Diversity of *D. opuntiae*-associated bacterial communities based on all developmental stages and locations. Constrained Analysis of Principal Coordinates (CAP) plot based on the Bray-Curtis metric (A) and the pairwise PERMANOVA comparison (B).
